# Supplementary material for: Goal-directed and flexible modulation of syllable sequence within birdsong
Source: Nat Commun. 2024 Apr 24;15:3419. doi: 10.1038/s41467-024-47824-1 (PMC11043396; doi:10.1038/s41467-024-47824-1)
Supplement: Supplementary file 1 — Supplementary Information [file 41467_2024_47824_MOESM1_ESM.pdf]

Supplementary Information

Goal-directed and flexible modulation of syllable sequence within birdsong

Takuto Kawaji, Mizuki Fujibayashi and Kentaro Abe

This pdf file contains:

Supplementary Figures 1-5
Supplementary Note

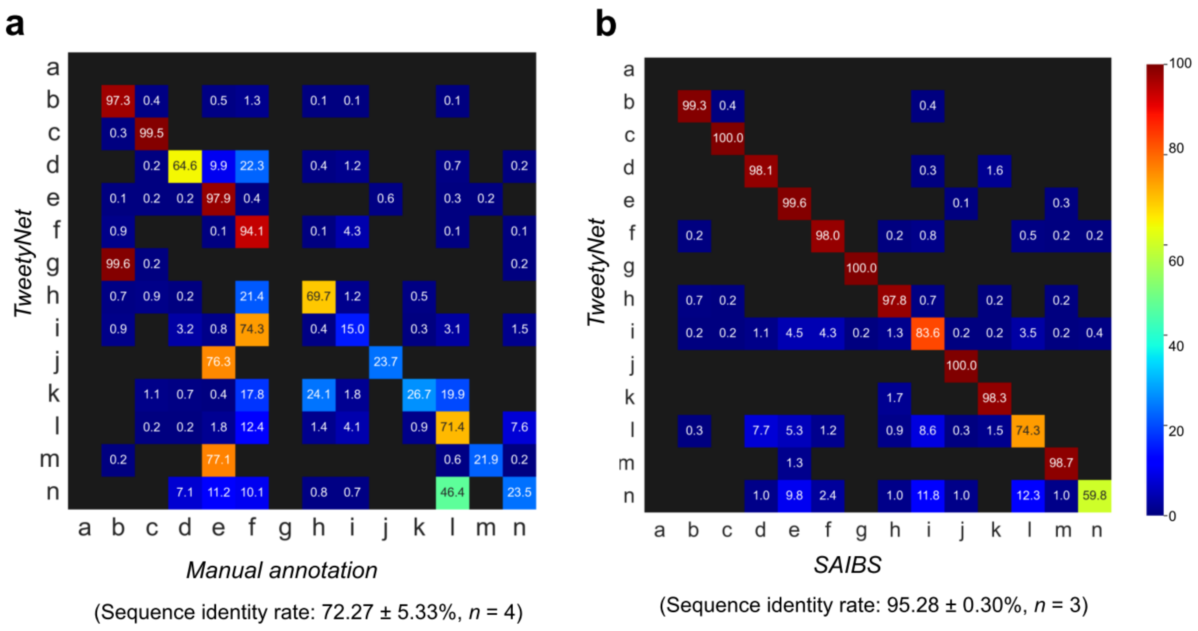

Supplementary Fig. 1. Comparison of manual and SAIBS annotation

a, b Comparison of syllable annotation between TweetyNet versus human (**a**) and TweetyNet versus SAIBS (**b**). Matrices show the mean syllable match rate (%) against TweetyNet, calculated from four skilled researchers and three independently trained SAIBS decoders. The same randomly selected 30 songs were used for analysis. The sequence identity rates of annotation are shown below.

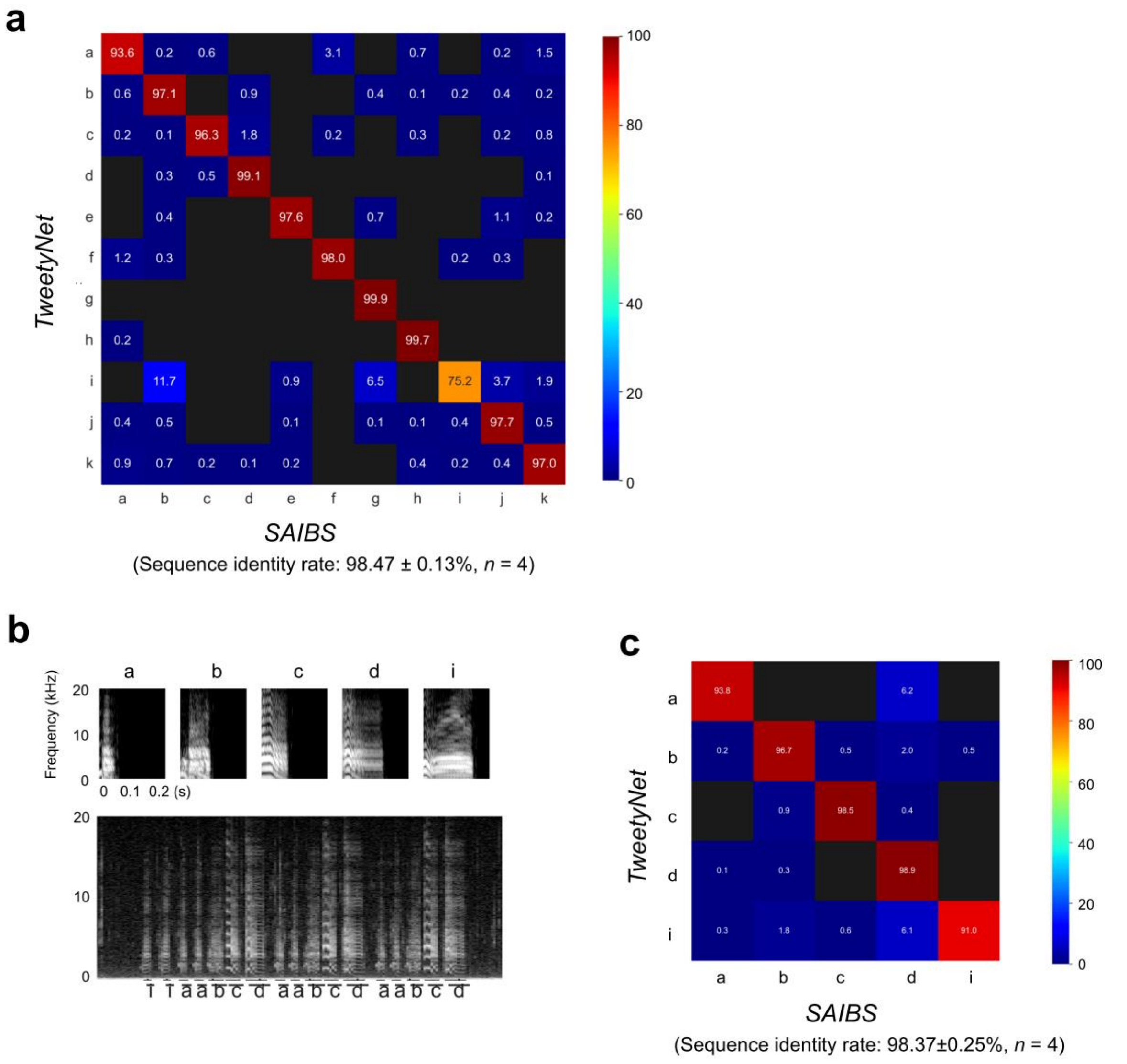

Supplementary Fig. 2. Annotation of Bengalese and zebra finch songs by SAIBS

a Annotation accuracy comparison against TweetyNet. Songs from another bird to the bird shown in Figure 1 were annotated by SAIBS, and the results were compared with TweetyNet. The matrix shows the mean syllable match rate (%), with the sequence identity rate of annotation shown below. Randomly selected 100 songs were used for analysis. Results from *n* = 4 trials are shown. **b** and **c** Example of syllables from one zebra finch automatically clustered by SAIBS (top), and their detection in a song bout (below). **c** Annotation accuracy comparison of zebra finch songs against TweetyNet, same as (**a**).

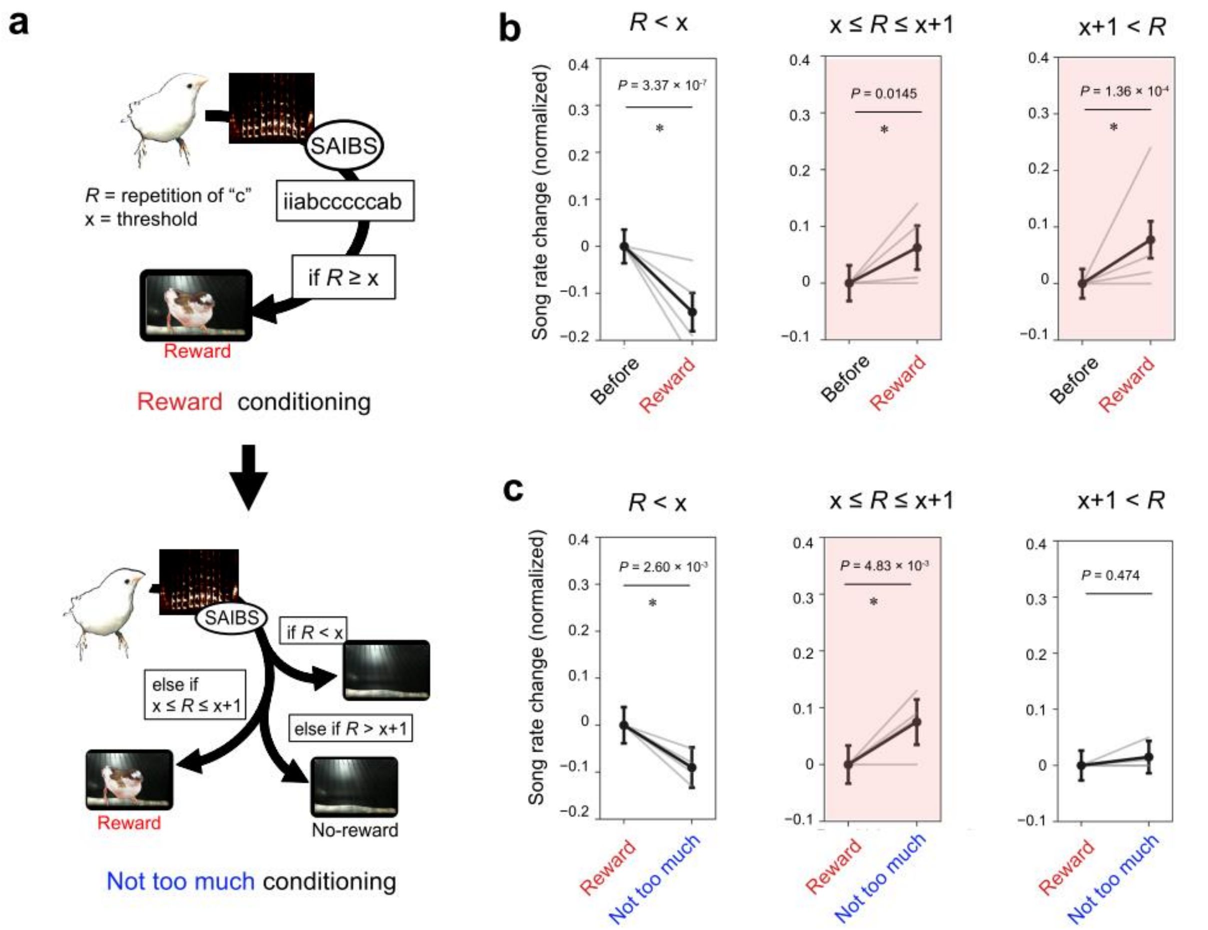

Supplementary Fig. 3. Flexible modulation of repetition range according to the reward

a Scheme of “Not-too-much” rule after the “Reward” conditioning. **b, c** Shift of song rate in three repetition range ($R <$ x, x $\leq R \leq$ x+1, x+1 $< R$) before and after the reward conditioning (**b**) and after “Not-too-much” conditioning (**c**). The reward ranges in each conditioning rules are highlighted in pink. Mean ± s.e.m., $n = 4$; P-values, paired t-test, $t(3) = 5.19, -2.46, -3.85$ (**b**), $t(3) = 3.03, -2.83, -0.72$ (**c**) for $R <$ x, x $\leq R \leq$ x+1, x+1 $< R$, respectively.

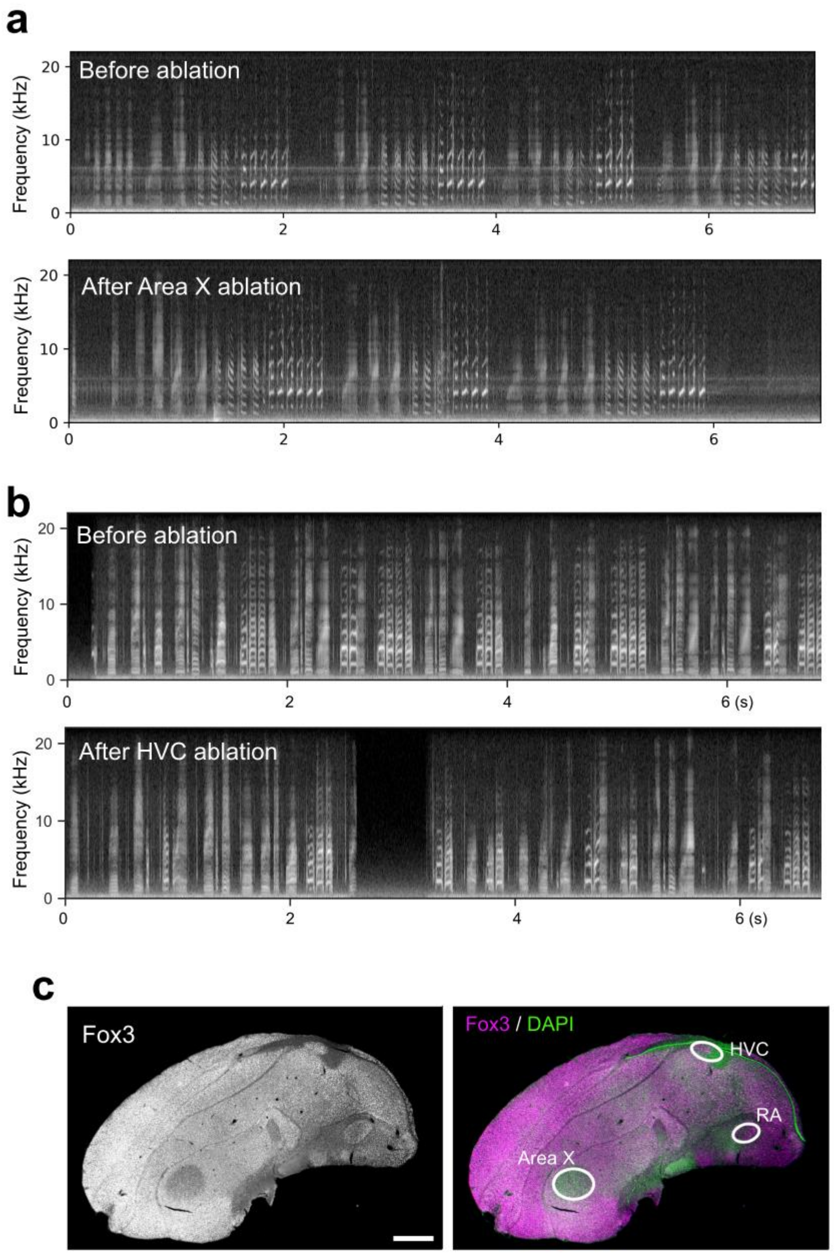

Supplementary Fig. 4. Effect of ablating HVC or Area X.

a, **b** Example of songs before and after ablation of Area X (**a**) or HVC (**b**). **c** A brain section of HVC ablated bird, immunostained against Fox3 (magenta), shown with DAPI (green). Scale bar, 1 mm.

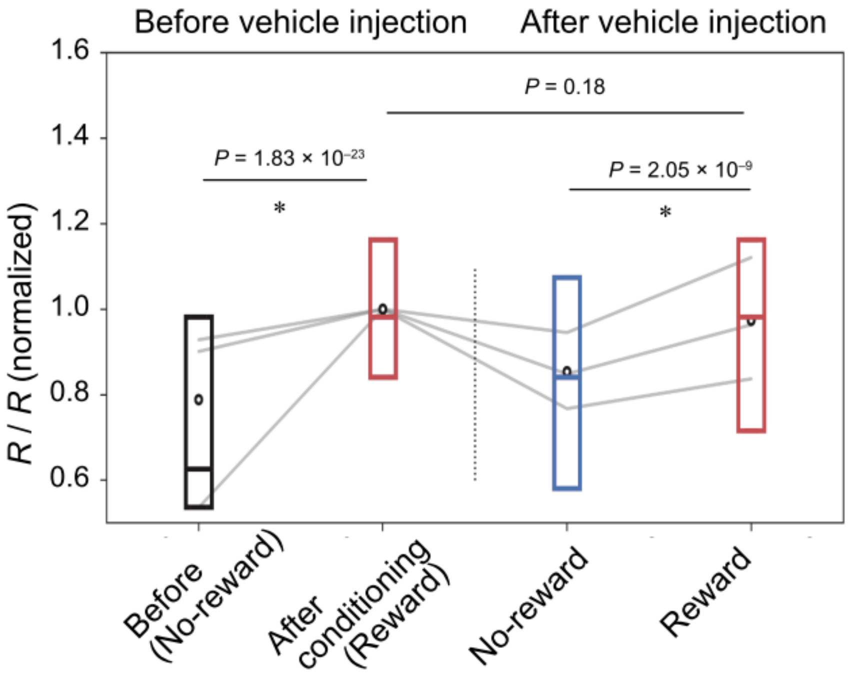

Supplementary Fig. 5. Effect of vehicle injection to Area X.

Syllable repetition before and after the vehicle (PBS) injection. The box plot shows the median and first and third quantiles, with the mean shown as a circle. *P*-values, Tukey's HSD-test, $t(299) = 10.9$, $t(299) = 1.35$, $t(299) = 6.18$ from left to right, $n = 3$ birds. We observed a significant difference between the vehicle and ibotenic acid injection regarding the context-depenendet modulation of songs post-injection (compared to the results shown in Fig. 5d; $P = 2.52 \times 10^{-7}$, LME model, $F(1199,1199) = 25.8$).

Supplementary Note

In this study, we introduced SAIBS, a novel syllable annotator for birdsong. Ideally, the accuracy of annotations should be evaluated against the “ground truth” of birdsong annotation. However, such a definitive “ground truth” is inherently unattainable for human observers through any objective methods. This is because of the subjective nature of communicative signals, which are best interpreted by the birds themselves. Thus, what the researchers in this field strive to do is to precisely match the spectral feature of the sound to specific syllable identities and label them as accurately and consistently as possible. Therefore, a direct comparison between manual labeling and SAIBS labeling is essentially futile, as neither approach can conclusively ascertain the “ground truth” of birdsong annotation. Accordingly, this study does not engage in assessing the accuracy or match rate of human versus SAIBS annotation. Instead, for quantitative and statistically compare the two methods, we adopt TweetyNet annotations as a hypothetical standard. Although not regarded as the absolute “ground truth”, TweetyNet serves as a benchmark for evaluating the precision and consistency of syllable annotations by both human and SAIBS. Given its acclaim for accuracy since its publication[1–3], TweetyNet is utilized as a comparator in this study. Our methodology involved contrasting the annotation accuracies of humans and SAIBS against those produced by TweetyNet. For this purpose, annotations automatically generated by SAIBS were employed to train TweetyNet. This "TweetyNet trained with SAIBS-generated annotations" served as our comparison standard, and compared the accuracy of “TweetyNet trained with SAIBS created annotation” versus manual annotation (Supplementary Fig. 1a) and “TweetyNet trained with SAIBS created annotation” versus SAIBS online decoder (Supplementary Fig 1b). The sequence identity rate for each researcher was compared to TweetyNet. The result from four researcher were 72.27 ± 5.33%. Then we performed the same process with three independently trained SAIBS and got the score of 95.28 ± 0.30%. This was statistically significant with $P = 4.84 \times 10^{-3}$ by Welch’s t-test. The primary reason for the lower accuracy in human annotations is due to the inherent variability in visual inspection-based annotations. This variability is evidenced by the higher deviation observed in human annotations compared to those by SAIBS, and significant less coefficient variance (SAIBS 0.317%, manual 7.38%). The results demonstrates that SAIBS is significant more accurate than manual annotation, positioning it on par with TweetyNet as an online decoder.

Supplementary References

1. Cohen, Y. *et al.* Automated annotation of birdsong with a neural network that segments spectrograms. *eLife* **11**, e63853 (2022).
2. Steinfath, E., Palacios-Muñoz, A., Rottschäfer, J. R., Yuezak, D. & Clemens, J. Fast and accurate annotation of acoustic signals with deep neural networks. *eLife* **10**, e68837 (2021).
3. Provost, K. L., Yang, J. & Carstens, B. C. The impacts of fine-tuning, phylogenetic distance, and sample size on big-data bioacoustics. *PLOS ONE* **17**, e0278522 (2022).
