# Supplementary material for: Goal-directed and flexible modulation of syllable sequence within birdsong
Source: Nat Commun. 2024 Apr 24;15:3419. doi: 10.1038/s41467-024-47824-1 (PMC11043396; doi:10.1038/s41467-024-47824-1)
Supplement: Supplementary file 3 — Reporting Summary [file 41467_2024_47824_MOESM3_ESM.pdf]

nature portfolio

Corresponding author(s): Kentaro Abe

Last updated by author(s): Mar 8, 2024

Reporting Summary

Nature Portfolio wishes to improve the reproducibility of the work that we publish. This form provides structure for consistency and transparency in reporting. For further information on Nature Portfolio policies, see our Editorial Policies and the Editorial Policy Checklist.

Statistics

For all statistical analyses, confirm that the following items are present in the figure legend, table legend, main text, or Methods section.

n/a	Confirmed	
☐	☒	The exact sample size (n) for each experimental group/condition, given as a discrete number and unit of measurement
☐	☒	A statement on whether measurements were taken from distinct samples or whether the same sample was measured repeatedly
☐	☒	The statistical test(s) used AND whether they are one- or two-sided<br>*Only common tests should be described solely by name; describe more complex techniques in the Methods section.*
☐	☒	A description of all covariates tested
☐	☒	A description of any assumptions or corrections, such as tests of normality and adjustment for multiple comparisons
☐	☒	A full description of the statistical parameters including central tendency (e.g. means) or other basic estimates (e.g. regression coefficient) AND variation (e.g. standard deviation) or associated estimates of uncertainty (e.g. confidence intervals)
☐	☒	For null hypothesis testing, the test statistic (e.g. F, t, r) with confidence intervals, effect sizes, degrees of freedom and P value noted<br>*Give P values as exact values whenever suitable.*
☒	☐	For Bayesian analysis, information on the choice of priors and Markov chain Monte Carlo settings
☒	☐	For hierarchical and complex designs, identification of the appropriate level for tests and full reporting of outcomes
☐	☒	Estimates of effect sizes (e.g. Cohen's d, Pearson's r), indicating how they were calculated

Our web collection on statistics for biologists contains articles on many of the points above.

Software and code

Policy information about availability of computer code

Data collection	Song data were acquired using Sound Analysis Pro 2011 (Tchernichovski et al., 2000) followed by further evaluation by SAIBS program executing on MATLAB R2020b (https://github.com/SAIBS-paper/SAIBS). Immunostained brain sections were acquired by BZ-9000 (Keyence).
Data analysis	Data was analyzed using Python 3.9.5. Immunostained brain sections were analyzed in Image J.

For manuscripts utilizing custom algorithms or software that are central to the research but not yet described in published literature, software must be made available to editors and reviewers. We strongly encourage code deposition in a community repository (e.g. GitHub). See the Nature Portfolio guidelines for submitting code & software for further information.

Data

Policy information about availability of data

All manuscripts must include a data availability statement. This statement should provide the following information, where applicable:

- Accession codes, unique identifiers, or web links for publicly available datasets
- A description of any restrictions on data availability
- For clinical datasets or third party data, please ensure that the statement adheres to our policy

Source data are provided with this paper. The code used in this study are available on GitHub: https://github.com/SAIBS-paper/SAIBS

Research involving human participants, their data, or biological material

Policy information about studies with human participants or human data. See also policy information about sex, gender (identity/presentation), and sexual orientation and race, ethnicity and racism.

Reporting on sex and gender	n/a
Reporting on race, ethnicity, or other socially relevant groupings	n/a
Population characteristics	n/a
Recruitment	n/a
Ethics oversight	n/a

Note that full information on the approval of the study protocol must also be provided in the manuscript.

Field-specific reporting

Please select the one below that is the best fit for your research. If you are not sure, read the appropriate sections before making your selection.

☒ Life sciences ☐ Behavioural & social sciences ☐ Ecological, evolutionary & environmental sciences

For a reference copy of the document with all sections, see nature.com/documents/nr-reporting-summary-flat.pdf

Life sciences study design

All studies must disclose on these points even when the disclosure is negative.

Sample size	Sample sizes were determined to be comparable to sample sizes in similar studies in this field.<br>For song analysis, 100 song samples acquired during the the designated period were evaluated with paired two-tailed t-test.
Data exclusions	No data were excluded from the analysis
Replication	All main results were repeated in multiple animals. The number of animals were indicated in the manuscript for each experiment. All findings were consistent across birds.
Randomization	Birds subjected to no conditioning (Fig 1d), non-social feedback (Fig 1e), nucleus ablation, and the "Frame color" conditioning were randomly allocated.
Blinding	Manual song annotation was performed by a researcher who was blind to the sample allocation.

Reporting for specific materials, systems and methods

We require information from authors about some types of materials, experimental systems and methods used in many studies. Here, indicate whether each material, system or method listed is relevant to your study. If you are not sure if a list item applies to your research, read the appropriate section before selecting a response.

Materials & experimental systems

n/a	Involved in the study	
☐	☒	Antibodies
☒	☐	Eukaryotic cell lines
☒	☐	Palaeontology and archaeology
☐	☒	Animals and other organisms
☒	☐	Clinical data
☒	☐	Dual use research of concern
☒	☐	Plants

Methods

n/a	Involved in the study	
☒	☐	ChIP-seq
☒	☐	Flow cytometry
☒	☐	MRI-based neuroimaging

Antibodies

Antibodies used	Mouse monoclonal anti-Fox3; MERCK; Cat#MAB377; RRID: AB_2298772

Validation	Validations are available on manufacture's website https://www.merckmillipore.com

Animals and other research organisms

Policy information about studies involving animals; ARRIVE guidelines recommended for reporting animal research, and Sex and Gender in Research

Laboratory animals	Bengalese Finch.
Wild animals	No wild animals were used in this study.
Reporting on sex	This study used only male birds.
Field-collected samples	No field collected samples were used in this study.
Ethics oversight	The care and experimental manipulation of animals used in this study were reviewed and approved by the Institutional Animal Care and Use Committee of Tohoku University.

Note that full information on the approval of the study protocol must also be provided in the manuscript.
