# Supplementary material for: Goal-directed and flexible modulation of syllable sequence within birdsong
Source: Nat Commun. 2024 Apr 24;15:3419. doi: 10.1038/s41467-024-47824-1 (PMC11043396; doi:10.1038/s41467-024-47824-1)
Supplement: Supplementary file 4 — Description of Additional Supplementary Files [file 41467_2024_47824_MOESM4_ESM.pdf]

Description of Additional Supplementary Files

Supplementary Movie 1

Description: Movie used for conditioning.

Supplementary Movie 2

Description: Movie used for non-social feedback, result of which is shown in Fig. 2e.
